# Greater family size is associated with less cancer risk: an ecological analysis of 178 countries

**DOI:** 10.1186/s12885-018-4837-0

**Published:** 2018-09-26

**Authors:** Wenpeng You, Frank J Rühli, Renata J Henneberg, Maciej Henneberg

**Affiliations:** 1 Adelaide Medical School, The University of Adelaide, Adelaide, SA 5005 Australia; 2 Institute of Evolutionary Medicine, University of Zurich, Zürich, Switzerland; 3 Biological Anthropology and Comparative Anatomy Unit, Adelaide Medical School, The University of Adelaide, Adelaide, Australia

**Keywords:** Total fertility rate, Household size, Psychological well-being, Family life, Cancer initiation

## Abstract

**Background:**

Greater family size measured with total fertility rate (TFR) and with household size, may offer more life satisfaction to the family members. Positive psychological well-being has been postulated to decrease cancer initiation risk. This ecological study aims to examine the worldwide correlation between family size, used as the measure of positive psychological well-being, and total cancer incidence rates.

**Methods:**

Country specific estimates obtained from United Nations agencies on total cancer incidence rates (total, female and male rates in age range 0–49 years and all ages respectively), all ages site cancer incidence (bladder, breast, cervix uteri, colorectum, corpus uteri, lung, ovary and stomach), TFR, household size, life expectancy, urbanization, per capita GDP PPP and self-calculated Biological State Index (I_bs_) were matched for data analysis. Pearson’s, non-parametric Spearman’s, partial correlations, independent T-test and multivariate regressions were conducted in SPSS.

**Results:**

Worldwide, TFR and household size were significantly and negatively correlated to all the cancer incidence variables. These correlations remained significant in partial correlation analysis when GDP, life expectancy, I_bs_ and urbanization were controlled for. TFR correlated to male cancer incidence rate (all ages) significantly stronger than it did to female cancer incidence rate (all ages) in both Pearson’s and partial correlations. Multivariate stepwise regression analysis indicated that TFR and household size were consistently significant predictors of all cancer incidence variables.

**Conclusions:**

Countries with greater family size have lower cancer risk in both females, and especially males. Our results seem to suggest that it may be worthwhile further examining correlations between family size and cancer risk in males and females through the cohort and case-control studies based on large samples.

**Electronic supplementary material:**

The online version of this article (10.1186/s12885-018-4837-0) contains supplementary material, which is available to authorized users.

## Background

Total fertility rate (TFR) representing the total number of births during a lifetime of a female [1, 2] has been used to measure childbearing and family size [3–5] in a number of studies. The prevalent conclusions were that more childbearing (greater TFR) may protect against female breast cancer [6, 7], corpus uteri cancer [8] and ovarian cancer [9] due to less oestrogen production or less menstrual cycles [10] and more oxytocin secretion [11, 12], but may contribute to cervix uteri cancer because of more exposure to infection risk [13].

The number of children born into a family does not only influence the mother’s physiological health of her reproductive system, but also has effects on health, including cancer development, and on her other systems and on all the other family members. For instance, greater family size has been postulated to protect family members from developing colorectal cancer [5], melanoma of skin [5], bladder cancer [5], breast cancer [5] and stomach cancer (in males only) [14]. Relationships between greater family size /household size and lung cancer [5, 15] and stomach cancer (females) [14] were explored, but without much success of seeing a clear trend. Aldrich et al. [15] reported that household size was in significant association with a risk of developing lung cancer in African Americans, but not in Latinos.

These controversial and circumstantial correlations between reproductive behaviour and a comprehensive health effect on all family members directed our attention to seeking alternative explanation of the relationship between TFR and risk factors for cancer. Psychological factors have been suggested to be linked with cancer initiation, but the mechanism has been intriguing professionals and laypeople for decades [16, 17]. Although studies on the possible effects of positive and negative psychological factors arising from life events on cancer incidence and prognosis are numerous, the literature remains contradictory as to methods and impacts [15, 18–20]. Extensive studies have suggested that adverse life events and the associated psychological stress may predispose to cancers in various body sites [21–25]. Everson et al. [26] reported that more stress may increase the cancer risk. This might be because people tend to recall adverse life events, but easily forget those positive ones, which constantly happen in the daily life. Cancer patients may more easily recall those negative life events which have been considered as cancer risks [27–31]. Only a limited number of studies have addressed the relationship between life satisfaction and cancer risk [15, 32], but the conclusions were controversial.

Research conducted into health effects of positive psychological well-being has concluded that family life satisfaction may stimulate oxytocin production in the human body [33–39], which may have the inhibitory effect on specific cancers [11, 12, 40, 41]. For example, positive psychological wellbeing has been postulated to protect against cancer risk in Israeli women [32], reduce the number of American cancer patients from going into metastasis [42] and help cancer patients with cancer’s detection, treatment, and survival [42]. Large families have greater life satisfaction in both Western and Eastern populations [43, 44]. Nan et al. [44] have also concluded that the bigger family size is, the higher Subjective Happiness Scale (SHS) result is in the family, regardless of cultural backgrounds.

Therefore, in this study, we assessed, from a global perspective, whether greater family size, measured with TFR [3] and household size may lower cancer risk using empirical, macro-level data obtained from international organizations.

## Methods

### Data sources

The population specific data were collected for this ecological study.

1. The GLOBOCAN 2012 estimates of incidence rates (age standardised, world) of all cancers excluding non-melanoma skin cancer (C00–97, but C44) in total, and separately for males and females of all ages [45] were used. Crude estimates of incidence rates of all cancers excluding non-melanoma skin cancer (C00–97, but C44) in total, and for males and females in age group 0–49 years were also obtained.
  The incidence rates of the individual site-specific cancers (bladder, breast, cervix uteri, colorectum, corpus uteri, lung, melanoma and ovary) were extracted as the dependent variables for data analysis in this study. The results from this study were aligned with the findings of previous studies of the relationships between family/household size and each of these site-specific cancers namely lung cancer [15], bladder cancer, melanoma and colon cancer [5].
  GLOBOCAN is a project conducted by the International Agency for Research on Cancer (IARC) of the World Health Organization. This project provides contemporary population level estimates by cancer site and sex using the best available data in each population and uses nine comprehensive methods of estimation [46].
2. The United Nations Statistics Division estimates of the life expectancy [47], the total population in households and the number of households [48].
  Life expectancy is the average number of years a person of a given age, residing in a given country is expected to live. We extracted the life expectancy at age 60 years (e_60_, 2005–2010) from abridged life Tables (1950–2100) [47] published online. Ageing has been a significant risk predictor of cancer. In this study, life expectancy (e_60_) was considered as the indicator of ageing.
  As instructed by the United Nations Statistics Division, we created a new variable, household size, through dividing the total population in households [48] by the number of households [48] in each country.
3. The World Bank published data [1] on Gross Domestic Product (GDP), total fertility rate (TFR) and urbanization.
  GDP was expressed in per capita purchasing power parity (PPP in current international $) in 2010. The World Bank also clusters countries into 4 classifications in terms of their GDP per capita (High Income, Upper Middle Income, Low Middle Income and Low Income). In this study, we grouped countries with High Income and Upper Middle Income as developed countries, and countries with Low Middle Income and Low Income as developing countries.
  Urbanization was expressed with the percentage of total population living in urban areas in 2010.
  Total Fertility Rate (TFR) represents total births per woman during her lifetime. It indicates the number of children that would be born to a woman if she were to live to the end of her childbearing years and bear children in accordance with age-specific fertility rates of the specified year.
  Total births per woman have been used to indicate the family size in studies at an individual level [3, 4]. Therefore, we used TFR as the measure of family size in this study, and terms “TFR” and “family size” were interchangeably used thereafter. Household size is used as the proxy of family size in this study that has been calculated from data independent from those used for TFR.
4. Biological State Index (I_bs_) was self-calculated [49, 50] with the fertility data of each country published by United Nations in 2008 [51] and the mortality data of life Tables (2009) published by World Health Organization (WHO) in 2012 [52].
  I_bs_ was included as one of the confounding factors that indicates the level of adaptation of a population [53]. The I_bs_ values range between 0 and 1.0. A greater I_bs_ value of a population means less opportunity for natural selection, and vice versa. The simplest interpretation of I_bs_ is that it indicates a probability with which an average person born into a population is able to pass her/his genes to the next generation. Recent studies have postulated that I_bs_ may indicate the magnitude of deleterious gene/mutation accumulation in a population due to relaxed natural selection [53–56]. The greater I_bs_ value means that a population has accumulated more deleterious gene/mutations of cancer [54], obesity [55] and type 1 diabetes [56], and vice versa [53–56]. Inclusion of I_bs_ as a confounder may remove the influence of cancer gene/mutation accumulation on the correlation between family size and cancer incidence.

### Data selection

We used country specific cancer incidence rates, TFR, GDP, urbanization, household size and life expectancies for all countries where the most updated and recent data were available (*N* = 178). In order to capture as many countries as we could for this study, we aligned country specific TFR with all cancer incidence rates, and then we matched other country-specific variables with the TFR.

Each country was treated as an individual subject in the analysis. Numbers of countries included in analyses of relationships with other variables may differ somewhat because all information was not uniformly available for all countries. The list of countries included in this study can be found in Additional file 1.

We singled out the population segment aged 0–49 years because females enter menopause at around 50 years of age and since then they produce less and less female hormones. Numerous studies have associated female oestrogen level with cancer risk [10, 11].

All the aforementioned data were freely available from the websites of the UN agencies. No ethical approval or written informed consent for participation was required.

### Data analysis

Scatter plots were produced in Excel (Microsoft® 2016) to explore and visualize the correlations between family size and cancer incidence rates for all ages in total population, males and females respectively. Scatterplots were repeated in the age group (0–49) for further testing the relationships between family size and cancer incidence rates. Scatter plots allowed us to assess data quality and distributions of variables. In the supplemental material, family size was replaced with household size for performing the scatter plots (Additional file 2).

Prior to correlation/regression analyses all data were log-transformed (ln) in order to reduce non-homoscedascity of their distributions and possible curvilinearity of regressions. To assess the relationships between each cancer incidence rate and family size, the analysis proceeded in four steps.

1. Pearson’s and nonparametric correlations (Spearman’s rho) were used to evaluate the strength and direction of the associations between family size and all other variables, including independent variables and confounders.
2. Partial correlation of Pearson’s moment-product approach was used to assess the relationship between each cancer incidence rate and family size respectively while we controlled for GDP PPP [45, 46], urbanization [57, 58], I_bs_ and life expectancy [59] which have been commonly considered as the contributing factors of cancer.
  Fisher’s r-to-z transformation was performed to test significance of differences between correlation coefficients.
3. Standard multiple linear regression (stepwise) was performed to identify the most significant predictor(s) of cancer risk. The dependent variables included cancer incidence rate by sex (total, male and female, age group 0–49 and all ages respectively). The independent variables/predictors entered into analyses were family size, urbanization, GDP, I_bs_ and life expectancy (not for cancer variables for 0–49 years).
4. The independent samples t-test was performed to compare the means of each cancer variable in high and low fertility countries divided at the cut point of TFR = 2.36. We used 2.36 as the cut point because it is the world average TFR published by the United Nations for the period of 2010–2015 [60].

Socioeconomic level plays a critical role in family happiness. In parallel to the analyses of the relationship between family size and cancer variables worldwide, the relationships between family size and each cancer variable in developed and developing country groupings were also examined respectively. Descriptive statistics including standard deviations of all variables were calculated for analysing and comparing the covariance (relationship between family size and cancer incidence) in all countries (*n* = 178), in developed world (*n* = 98) and in developing world (*n* = 80).

Subsequently, family size was substituted with household size for reanalysing the associations and regressions. The results were reported in Additional files 3 and 4 (Tables S2 and S3). There was no stratification of country grouping in the supplemental analyses due to limited sample size of countries for which household size was available (*n* = 58).

Pearson’s, non-parametric Spearman’s rho correlations, partial correlation, stepwise multiple linear regression, independent samples *t*-test analyses and descriptive statistics were calculated using SPSS v. 22 (SPSS Inc., Chicago Il USA). To increase homoscedasticity of data distributions, log-transformed variables were used for correlation analyses. The significance was reported when *P*-value was < 0.05, but the significance levels of *p* < 0.01 and *p* < 0.001 were also indicated in the tables. Regression analysis criteria were set at probability of F to enter ≤0.05 and probability of F to remove ≥0.10. The raw data were used for scatter plots.

## Results

**Fig. 1:**
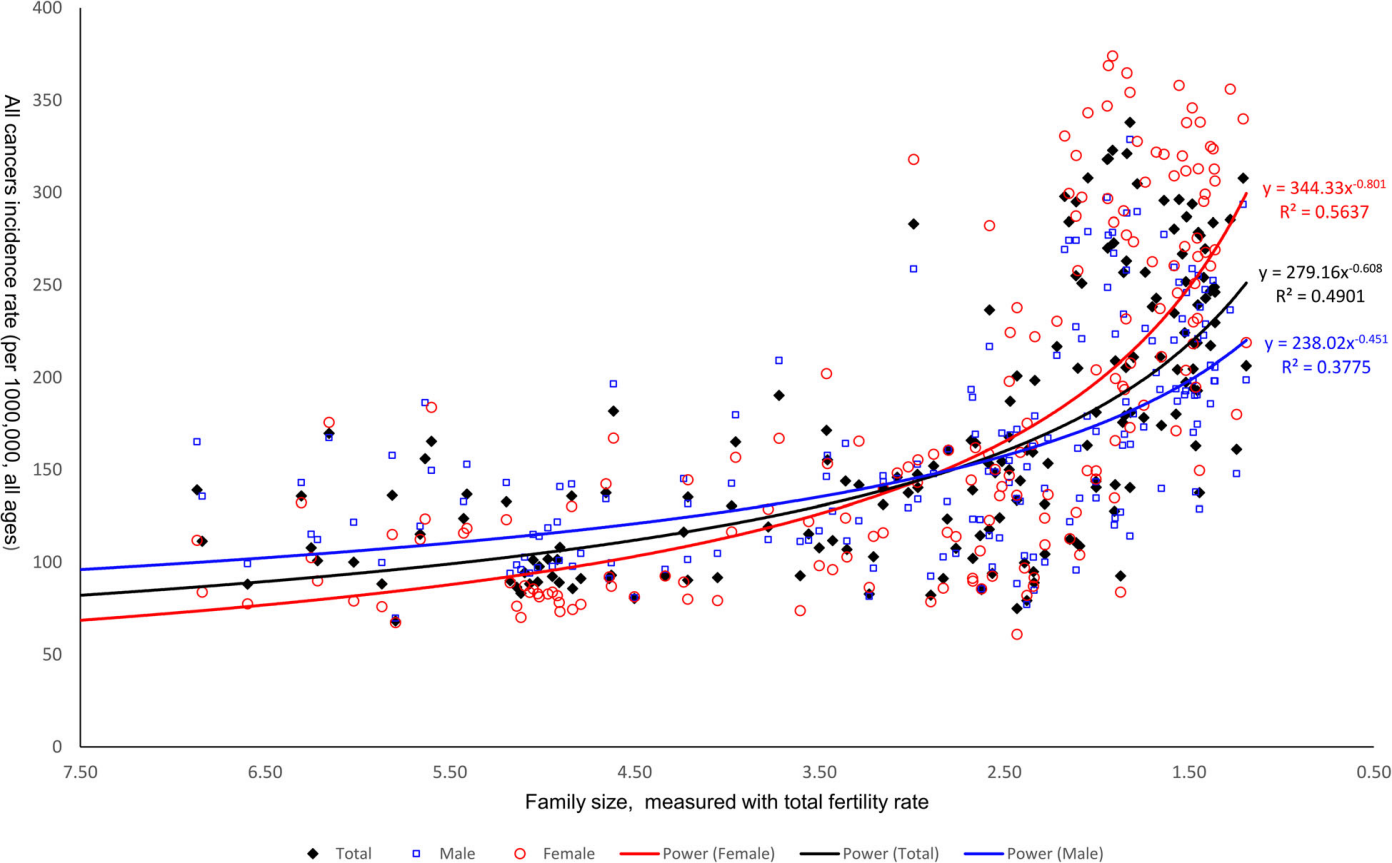
The relationships between family size and all cancers incidence rates (total, male and female, all ages)

**Fig. 2:**
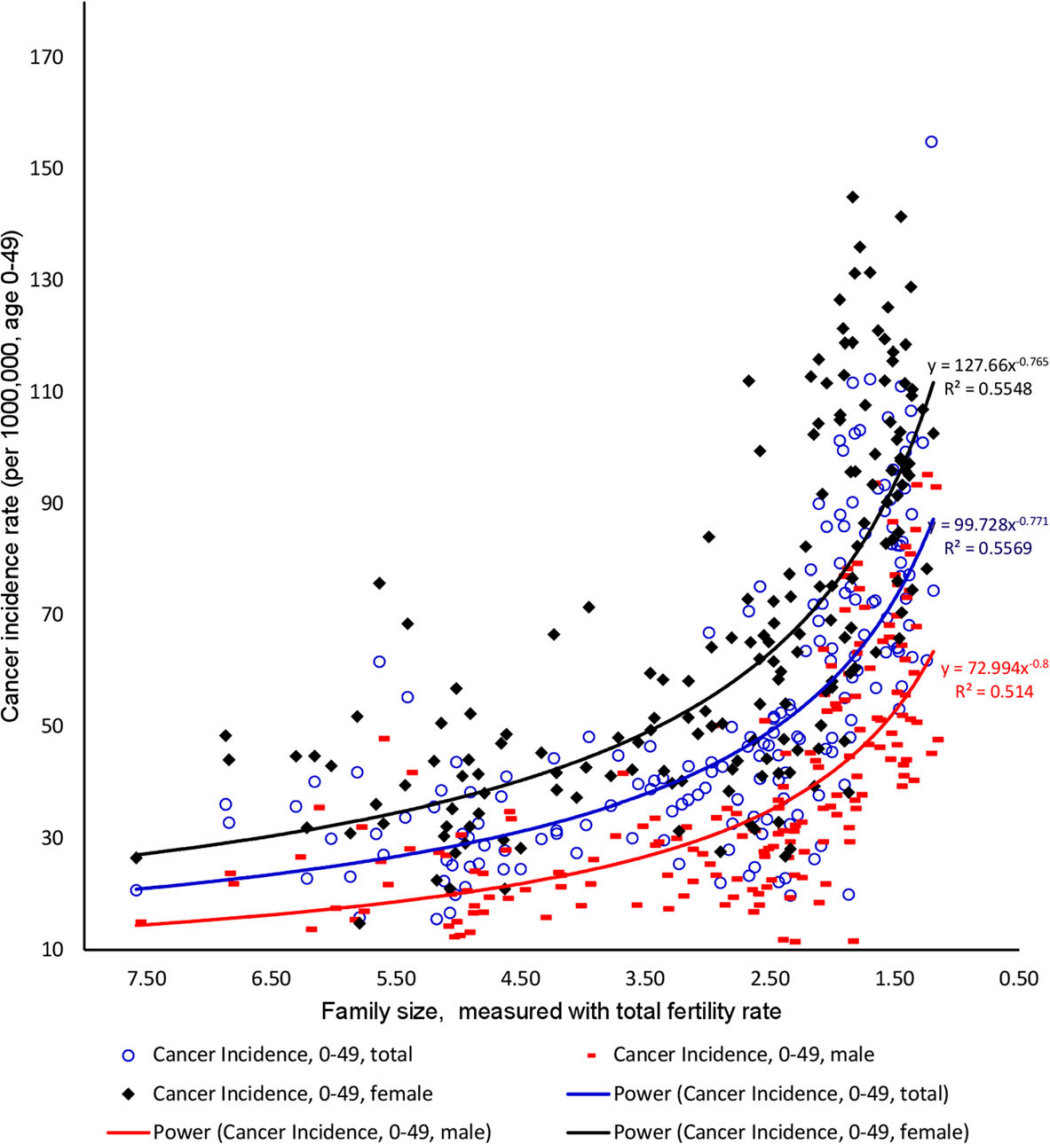
The relationships between family size and all cancers incidence rates (total, male and female, age 0–49)

Figure 1 shows a negative and strong correlation of family size to cancer incidence rates in total population and in males and females separately (all ages). The non-linear relationships between family size and group cancer incidence variables (total population, males, females in all ages) identified in the scatterplots show the strong correlation between family size and cancer incidence rate (R^2^ = 0.4901, 0.3755 and 0.5637 respectively). The relationships are also true in the age (0–49) group (Fig. 2). Household size as the proxy of family size has shown the similar correlation to all cancers incidence rates (total, female and male) (Additional file 1: Figure S1).

The subsequent analyses of log-transformed data proved these relationships. Globally (*n* = 178), Spearman’s rank correlation showed that family size was in significant negative correlation to all cancers incidence rates (both sexes) in all ages (*r* = − 0.716, *p* < 0.001) and in age group 0–49 years (*r* = − 0.752, *p* < 0.001), separately in females of all ages (*r* = − 0.640, *p* < 0.001) and age group 0–49 years (*r* = − 0.762, *p* < 0.001) and in males of all ages (*r* = − 0.761, *p* < 0.001) and age group 0–49 years (*r* = − 0.765, *p* < 0.001) (Table 1). Pearson’s *r* showed quite similar relationship trends between family size and the cancer variables (Table 1).

**Table 1:** Pearson, Nonparametric and partial correlation between family size and each cancer variable

	All countries *n* = 178						Developed countries *n* = 98								Developing countries *n* = 80			
	Pearson		Nonparametric		Partial^		Pearson		Nonparametric		Partial^		Pearson		Nonparametric		Partial^	
	r	n	rho	n	r	df	r	n	rho	n	r	df	r	n	rho	n	r	df
All cancers excl. Non-melanoma skin cancer (C00–97, but C44) - all ages: total	−0.700^***^	178	−0.716^***^	178	− 0.362^***^	161	− 0.625^***^	98	− 0.540^***^	98	− 0.470^***^	87	− 0.399^***^	80	− 0.334^**^	80	−0.172	68
All cancers excl. Non-melanoma skin cancer (C00–97, but C44)- all ages: female	−0.614^***^	178	−0.640^***^	178	−0.230^**^	161	−0.559^**^	98	−0.477^***^	98	−0.362^***^	87	−0.200^*^	80	−0.140	80	−0.018	68
All cancers excl. Non-melanoma skin cancer (C00–97, but C44) - all ages: male	−0.751^***^	178	−0.761^***^	178	−0.449^***^	161	−0.661^***^	98	−0.582^***^	98	−0.532^***^	87	−0.530^***^	80	−0.457^***^	80	−0.303^*^	68
All cancers excl. Non-melanoma skin cancer (C00–97, but C44) – 0-49: total^a^	− 0.752^***^	178	−0.777^***^	178	−0.534^***^	162	−0.658^***^	98	−0.655^***^	98	−0.581^***^	88	−0.563^***^	80	−0.498^***^	80	−0.430^***^	69
All cancers excl. Non-melanoma skin cancer (C00–97, but C44) – 0-49: female^a^	− 0.751^***^	178	−0.762^***^	178	−0.492^***^	162	−0.633^***^	98	−0.581^***^	98	−0.534^***^	88	−0.537^***^	80	−0.482^***^	80	−0.384^***^	69
All cancers excl. Non-melanoma skin cancer (C00–97, but C44) – 0-49: male^a^	− 0.727^***^	178	−0.765^***^	178	−0.542^***^	162	−0.684^***^	98	−0.705^***^	98	−0.621^***^	88	−0.526^***^	80	−0.449^***^	80	−0.430^***^	69
Bladder (C67), all ages	−0.622^***^	178	−0.653^***^	178	−0.250^***^	161	−0.532^***^	98	−0.552^***^	98	−0.406^***^	87	−0.253^*^	80	−0.144	80	−0.112	68
Breast(C50), all ages	−0.565^***^	178	−0.626^***^	178	−0.088	161	−0.475^***^	98	−0.406^***^	98	−0.213^*^	87	−0.077	80	−0.102	80	0.091	68
Cervix uteri (C53), all ages	0.451^***^	178	0.504^***^	178	−0.110	161	0.192	98	0.223^*^	98	−0.067	87	0.358^***^	80	0.470^***^	80	−0.038	68
Colorectum (C18–21), all ages	−0.777^***^	178	−0.790^***^	178	−0.316^***^	161	−0.698^***^	98	−0.638^***^	98	−0.528^***^	87	−0.489^***^	80	−0.436^***^	80	−0.091	68
Corpus uteri (C54), all ages	−0.599^***^	177	−0.639^***^	177	−0.251^***^	161	−0.477^***^	98	−0.441^***^	98	−0.328^**^	87	−0.309^**^	79	−0.258^*^	79	−0.095	68
Lung (C33–34), all ages	−0.829^***^	178	−0.798^***^	178	−0.453^***^	161	−0.650^***^	98	−0.576^***^	98	−0.525^***^	87	−0.748^***^	80	−0.725^***^	80	−0.442^***^	68
Melanoma of skin (C43), all ages	−0.393^***^	173	−0.405^***^	173	−0.103	159	−0.373^***^	94	−0.334^***^	94	−0.192	84	0.297^**^	79	0.353^***^	79	0.145	68
Ovary (C56), all ages	−0.551^***^	178	−0.609^***^	178	−0.282^***^	161	−0.564^***^	98	−0.563^***^	98	−0.444^***^	87	−0.198	80	−0.182	80	−0.089	68
Stomach (C16), all ages	−0.504^***^	178	−0.527^***^	178	−0.301^***^	161	−0.410^***^	98	−0.376^***^	98	−0.350^***^	87	−0.496^***^	80	−0.460^***^	80	−0.223	68
GDP PPP 2010	−0.714^***^	178	−0.703^***^	178	–	–	−0.418^***^	98	−0.331^***^	98	–	–	−0.579^***^	80	−0.644^***^	80	–	–
Urbanization 2010	−0.772^***^	170	−0.742^***^	170	–	–	−0.278^**^	94	−0.254^*^	94	–	–	−0.656^***^	76	−0.650^***^	76	–	–
Life Expectancy (e_60_, 2005–2010)	−0.580^***^	178	−0.571^***^	178	–	–	−0.155	98	−0.043	98	–	–	−0.357^***^	80	−0.336^**^	80	–	–
Biological State Index (I_bs_)	−0.818^***^	172	−0.850^***^	172	–	–	−0.577^***^	94	−0.593^***^	94	–	–	−0.767^***^	78	−0.826^***^	78	–	–

Note: Pearson, Nonparametric and partial correlation reported. Significance level: ***p < 0.001, **p < 0.01, *p < 0.05

^Partial correlations were calculated when GDP, Urbanization, Life expectancy (e60) and Biological State Index (I_bs_) were kept statistically constant

^a^Life expectancy (e_50_) was not controlled for as it is not relevant in population segment aged 0–49 years old

Data sources and variable meanings:

The International Agency for Research published cancer incidence rates (per 100,000 in 2012) of all cancers incidence rate by sex (total, male and female, 0–49 years and all ages respectively); bladder, breast, cervix uteri, colorectum, corpus uteri, ovary and tomach

The World Bank data: Total Fertility Rate (the mean number of children born to a woman between 2009 and 2011), GDP PPP (per capita purchasing power parity in current international $ in 2010) and Urbanization (the percentage of total population living in urban areas in 2010), Income classifications (High, Upper Middle, Low Middle, Low) used to stratified the 178 countries

The United Nations data: Life expectancy (e_60_, 2005–2010)

United Nations published (2008) country specific fertility data and WHO published (2012) life table were used for calculating the Biological State Index (I_bs_)

All variables were log-transformed for analysis in SPSS

When family size was replaced with household size for supplemental data analysis, household size also showed significant, negative and strong correlation to each cancer variable (both sexes, female and male in age groups, 0–49 and all ages respectively) (Additional file 3: Table S1).

In developed countries grouping (*n* = 98), Spearman’s rank correlation showed that family size was in significant negative correlation to all cancers incidence rates (both sexes) in all ages (*r* = − 0.540, *p* < 0.001) and age group 0–49 years (*r* = − 0.705, *p* < 0.001), separately in females of all ages (*r* = − 0.477, *p* < 0.001) and age group 0–49 years (*r* = − 0.581, *p* < 0.001) (Table 1) and in males of all ages (*r* = − 0.582, *p* < 0.001) and age group 0–49 years (*r* = − 0.705, *p* < 0.001). Pearson’s *r* showed quite similar relationship trends between family size and the cancer variables (Table 1).

In developing countries grouping (*n* = 80), Spearman’s rank correlation showed that family size was in significant negative correlation to all cancers incidence rates in all ages (*r* = − 0.334, *p* < 0.001) and age group 0–49 years (*r* = − 0.498, *p* < 0.001), separately in females of age group 0–49 years (*r* = − 0.482, *p* < 0.001) but not at all ages (*r* = − 0.140) and in males of all ages (*r* = − 0.457, *p* < 0.001) and age group 0–49 years (*r* = − 0.449, *p* < 0.001) (Table 1). Pearson’s *r* showed quite similar relationship trends between family size and the cancer variables, except for all cancers incidence rate in females at all ages (*r* = − 0.200, *r* < 0.05) (Table 1).

When, in the partial correlation analyses, we controlled for the major confounders (GDP, urbanization, life expectancy (not for cancer variable in age group 0–49 years) and I_bs_): 1) globally (*n* = 178), family size remained in the significant correlation to all cancer incidence rates (both sexes) in all ages (*r* = − 0.362, *p* < 0.001) and the age group 0–49 years (*r* = − 0.534, *p* < 0.001), in females of all ages (*r* = − 0.230 *p* < 0.001) and age group 0–49 years (*r* = − 0.492, *p* < 0.001) and in males of all ages (r = − 0.449, *p* < 0.001) and age group 0–49 years (*r* = − 0.542, *p* < 0.001) (Table 1). Family size correlated stronger with male cancers incidence than with female cancers in all ages group (*n* = 178). This difference was shown to be statistically significant by Fisher’s r-to-z transformation in both Pearson’s (z = 2.43, *p* = 0.015) and partial (z = 2.22, *p* = 0.026) correlations. 2) In developed world (*n* = 98), family size also remained in the significant correlation to all cancers incidence rates (both sexes) in all ages (*r* = − 0.625, *p* < 0.001) and the age group 0–49 years (*r* = − 0.658, *p* < 0.001), in males of all ages (*r* = − 0.470, *p* < 0.001) and age group 0–49 years (*r* = − 0.581, *p* < 0.001) and in females of all ages (r = − 0.362, *p* < 0.001) and the age group 0–49 years (r = − 0.534, *p* < 0.001) (Table 1). 3) In developing world (*n* = 80), family size remained in the significant correlation to all cancer incidence rates (both sexes) in the age group 0–49 years (*r* = − 0.430, *p* < 0.001) but not at all ages group, in females of age group 0–49 years (*r* = − 0.384, p < 0.001) but not at all ages and in males of all ages (*r* = − 0.303, *p* < 0.05) and the age group 0–49 years (r = − 0.430, *p* < 0.001) (Table 1).

Table 1 also shows that, globally (*n* = 178) and in developed world (*n* = 98), each of the incidence rates (all ages) of individual site cancers in bladder, breast, colorectum, corpus uteri, lung, skin (melanoma), ovary and stomach was in significant, negative and strong correlation to family size in both Pearson’s and partial correlation analyses (Table 1). Globally (*n* = 178), cervix uteri cancer correlated with family size significantly and positively in both Pearson’s r and non-parametric correlation, but the correlation was neither strong nor significant in partial correlation (Table 1). In developed world (*n* = 80), cervix uteri cancer did not show correlation (partial) or very weak correlation (Pearson’s r) with family size (Table 1) although it statistically significantly correlated with family size (*r* = 0.223, *p* < 0.05). In developing world (*n* = 80), only correlations between family size and lung cancer and cervix uteri cancer were consistent with those revealed globally and in developed world (Table 1).

The correlations, especially the partial correlations between family size and cancer variables in all countries (*n* = 178) and in developed world (*n* = 98) were stronger and more significant than those in developing world. Variances of cancer incidence variables in developing world (*n* = 80) were smaller than their counterparts in the developed world and all countries grouping (Additional file 5).

Table 2 shows that, globally (*n* = 178), the mean incidence rate of each cancer variable in country group (*n* = 95) with TFR ≥ 2.36 was significantly (*p* < 0.001) lower than that of country group (*n* = 83) with TFR < 2.36 except cervix uteri cancer. This trend remained in the developed country grouping (*n* = 98) except cervix uteri and stomach cancers, and in developing grouping (*n* = 80) except cancers in breast, cervix uteri, melanoma (skin) and ovary.

**Table 2:** Independent samples t test to compare the differences between the means in two country groups with the cut point of 2.36 (TFR). Sample size 95 indicates countries with high TFR, 83 low TFR

	All countries, *n* = 178				Developed Countries, *n* = 98				Developing Countries, *n* = 80			
	Sample size	Mean	Mean difference	t	Sample size	Mean	Mean difference	t	Sample size	Mean	Mean difference	t
All cancers excl. Non-melanoma skin cancer (C00–97, but C44) - all ages: both sexes	95	123.81	−96.58	−12.50^***^	26	141.57	−88.17	−6.67^***^	69	117.11	−42.01	−3.89^***^
	83	220.39			72				11			
All cancers excl. Non-melanoma skin cancer (C00–97, but C44)- all ages: female	95	129.73	−72.58	−10.67^***^	26	143.80	−66.91	−5.88^***^	69	124.42	−22.87	−2.23^*^
	83	202.31			72				11			
All cancers excl. Non-melanoma skin cancer (C00–97, but C44) - all ages: male	95	120.09	− 128.54	−13.45^***^	26	144.41	− 114.97	−7.00^***^	69	110.93	−67.41	−5.03^***^
	83	248.64			72				11			
All cancers excl. Non-melanoma skin cancer (C00–97, but C44) – 0-49: both sexes	95	35.98	−36.82	−13.37^***^	26	43.04	−32.18	−6.49^***^	69	33.32	−23.58	−6.73^***^
	83	72.80			72				11			
All cancers excl. Non-melanoma skin cancer (C00–97, but C44) – 0-49: female	95	47.01	−45.90	−12.87^***^	26	58.15	−38.23	−6.06^***^	69	42.82	−27.40	−6.06^***^
	83	92.91			72				11			
All cancers excl. Non-melanoma skin cancer (C00–97, but C44) – 0-49: male	95	25.24	−28.28	−12.68^***^	26	28.57	−26.50	−6.52^***^	69	23.99	−19.38	−6.41^***^
	83	53.52			72				11			
Bladder (C67), all ages	95	2.91	−4.76	−9.48^***^	26	4.03	−4.12	− 4.75^***^	69	2.49	−2.03	−2.70^**^
	83	7.67			72				11			
Breast (C50), all ages	95	31.01	−29.29	−10.43^***^	26	38.65	−25.47	−5.46^***^	69	28.13	−7.15	−1.80
	83	60.30			72				11			
Cervix uteri (C53), all ages	95	26.18	13.24	7.17^***^	26	20.85	8.29	4.02^***^	69	28.19	12.77	2.61^*^
	83	12.94			72				11			
Colorectum (C18–21), all ages	95	7.63	−16.04	−13.12^***^	26	11.34	−13.91	−6.68^***^	69	6.24	−7.11	−4.51^***^
	83	23.67			72				11			
Corpus uteri (C54), all ages	95	5.15	−6.91	−8.91^***^	26	7.23	−5.21	−4.06^***^	69	4.37	−5.24	−3.76^***^
	83	12.06			72				11			
Lung (C33–34), all ages	95	7.43	−17.12	−12.62^***^	26	11.42	−13.76	−6.16^***^	69	5.93	−14.55	−5.94^***^
	83	24.55			72				11			
Melanoma of skin (C43), all ages	95	1.16	−4.80	−6.35^***^	26	1.67	−5.05	−3.38^***^	69	0.97	−0.07	−0.25
	83	5.96			72				11			
Ovary (C56), all ages	95	4.67	−3.39	−9.27^***^	26	5.09	−3.31	−5.56^***^	69	4.51	−1.28	−1.95
	83	8.06			72				11			
Stomach (C16), all ages	95	6.29	−3.32	−3.71^***^	26	7.24	−2.10	−1.50	69	5.92	−5.38	−2.90^**^
	83	9.61			72				11			

Note: Significance level: ***p < 0.001, **p < 0.01, *p < 0.05

Data sources and variable meanings:

The International Agency for Research published cancer incidence rates (per 100,000 in 2012) of all cancers incidence rate by sex (total, male and female, 0–49 years and all ages respectively); bladder, breast, cervix uteri, colorectum, corpus uteri, ovary and stomach

The World Bank data: Total Fertility Rate (TFR, the mean number of children born to a woman between 2009 and 2011). The TFR 2.36 was used as the cut point to stratify the total countries (*n* = 178)

For each cancer variable, the mean of countries with TRR ≥2.36 was reported in the first line, and the mean of countries with TFR < 2.36 was reported in the second line. Sample sizes: *n* = 95 and 83

In the standard multiple linear (stepwise) regression analyses, family size was the significant predictor of the total, male and female cancer incidence rates (with exception of all female cancers at all ages) in samples of all ages and in age group 0–49 years respectively when family size, GDP, life expectancy (not for age group 0–49 years) and I_bs_ were entered as the independent variables/predictors (Table 3). Although family size is a significant predictor of the variable of all cancers in females at all ages, the value of its beta coefficient was smaller than for the variable of all cancers in males at all ages. This finding was consistent with those reported in Table 1 that family size was in significantly stronger negative association with all cancers in males at all ages than it was with all cancers in females at all ages in both Pearson’s (z = 2.43, *p* = 0.015) and partial (z = 2.22, *p* = 0.026) correlation analyses. This means that greater family size may have more protective effects on male cancer risk than on female (cancer risk.

**Table 3:** Stepwise multiple linear regression to identify the significant predictors of cancer incidence risk

	All countries *n* = 178				Developed countries (High Quality Data)*n* = 65			Developing countries (Low Quality Data) *n* = 113		
	Rank	Predictor	Beta	Adjusted R^2^	Predictor	Beta	Adjusted R^2^	Predictor	Beta	Adjusted R^2^
All cancers excl. Non-melanoma skin cancer (C00–97, but C44) - all ages: both sexes	1	Family Size	−0.473***	0.502	Family Size	−0.503***	0.403	Family Size	−0.396***	0.145
	2	Life Expectancy	0.330***	0.552	Life Expectancy	0.327***	0.487	^	^	^
All cancers excl. Non-melanoma skin cancer (C00–97, but C44)- all ages: female	1	Life Expectancy	0.385***	0.402	Family Size	−0.424***	0.320	Life Expectancy	0.296*	0.075
	2	Family Size	−0.351***	0.459	Life Expectancy	0.353***	0.417	^	^	^
All cancers excl. Non-melanoma skin cancer (C00–97, but C44) - all ages: male	1	Family Size	− 0.576***	0.572	Family Size	−0.563***	0.450	Family Size	−0.522***	0.262
	2	Life Expectancy	0.254***	0.601	Life Expectancy	0.268***	0.505	^	^	^
All cancers excl. Non-melanoma skin cancer (C00–97, but C44) – 0-49: both sexes^a^	1	Family Size	−0.754***	0.567	Family Size	−0.662***	0.432	Family Size	−0.640 ***	0.296
	2	^	^	^	^	^	^	Urbanization	−0.246*	0.340
All cancers excl. Non-melanoma skin cancer (C00–97, but C44) – 0-49: female^a^	1	Family Size	− 0.755***	0.568	Family Size	−0.641***	0.404	Family Size	−0.531***	0.272
All cancers excl. Non-melanoma skin cancer (C00–97, but C44) – 0-49: male^a^	1	Family Size	−0.730***	0.530	Family Size	−0.685***	0.464	Family Size	−0.610***	0.247
	2	^	^	^	^	^	^	Urbanization	−0.291**	0.312

Note: Significance level: ***p < 0.001, **p < 0.01, *p < 0.05

Variables (log-transformed) entered for multiple linear regression (stepwise) analysis: Family Size, Life Expectancy (e_60_), GDP PPP, Urbanization and Biological State Index (I_bs_)

^a^Life expectancy (e_50_) was not included as it is not relevant in population segment aged 0–49 years

^ No other variable identified as the significant predictor

Data sources and variable meanings:

The International Agency for Research published cancer incidence rates (per 100,000 in 2012) of all cancers incidence rate by sex (total, male and female, 0–49 years and all ages respectively); bladder, breast, cervix uteri, colorectum, corpus uteri, ovary and stomach

The World Bank data: Total Fertility Rate (between 2009 and 2011), GDP PPP (per capita purchasing power parity in current international $ in 2010) and Urbanization (the percentage of total population living in urban areas in 2010)

The United Nations data: Life expectancy (e_60_, 2005–2010), the total population in households and the number of households for calculating household size

United Nations published (2008) country specific fertility data and WHO published (2012) life tables were used for calculating the Biological State Index (I_bs_)

Comparing with those correlations in all countries and in developed world, correlations between family size and cancer variables became weak and/or insignificant in the developing country grouping when standard deviation of cancer variable became low (Additional file 4: Table S2).

As the proxy of family size, household size was identified as the significant predictor of the total, male and female cancer incidence rates in samples of all ages and in age group 0–49 years respectively when household size, GDP, urbanisation, life expectancy (not for age group 0–49 years) and I_bs_ were entered as the independent variables/predictors in the standard multiple linear (stepwise) regression analysis (Additional file 5: Table S3).

## Discussion

Cancer risk has been associated with multiple aetiologies, which may act through various mechanisms. Our results showed that: 1) Worldwide, smaller family size may be an independent determinant of increased cancer risk. 2) increased family size may show more protecting effects on cancer risk in males than females.

It is necessary to note the limitations of our work before analysing the public health implications of this study:

First, the observational data were used in our work, which makes the results subject to inherent bias, i.e. “correlation between two variables does not mean causality”.

Second, we must highlight the ecological fallacy (intrinsic limitation) arising from the ecological study approach which was adopted in this study. The data included in this study were calculated for country/populations as a whole. Thus, values for risk-modifying factors do not always hold true for individuals to predict their cancer risk. However, we would like to note that it is nearly impossible to test the relationships at the individual family level due to rare occurrence rate of cancers, and even rarer in some individual site-specific cancers, such as ovarian cancer.

Finally, data compiled and/or collected by the major international agencies (WHO, IARC, the United Nations and the World Bank) might be crude, and may contain some random errors arising from methods of reporting incidence of specific diseases, reliability of diagnoses and possible administrative errors.

Despite these limitations, findings from different data analyses in this study consistently show that country with greater TFR (family size) has lower cancer incidence rate regardless of age range and sex. This relationship trend has been observed in the correlations between family size and not only the individual site cancers, but also all cancers (in males, females and both sexes). The broad correlations between family size and cancers expressed in different individual sites, sexes and groups, may not be simply explained by the female hormonal fluctuation due to pregnancy and breastfeeding.

The relationship between psychological well-being and diseases (body and mind) has been an old issue. In the past, research into well-being has mainly focused on negative attitudes and affects. The majority of the studies documented that negative life events (death, divorce, injury, car crash etc.,), stressful life style, depression and/or anxiety, may lead to developing cancers [32, 61]. However, there is a documented bias in the data collected from the individual based surveys. In general, cancer patients tend to report negative events in excess compared to other people with average or positive attitudes [28, 30, 31]. This has been reported or reflected in a number of studies [27–30] regarding the relationship between cancer risk and adverse life events. According to the ancient Chinese medicine textbook, which was compiled 2200 years ago, it has been believed that people have five internal organs of five gases (five emotions), i.e. happiness, anger, sadness, worry and fear. Among these five gases, only happiness makes the gas smooth [62], which keeps people healthy.

Family has long been cited as a health promoting factor [63, 64], and family size has been associated with life satisfaction [43, 44, 65]. From the perspective of evolution, humans have adapted early to cooperative breeding [66, 67], and then evolved alloparental care [68], and biological foundations of such human love may be heritable generation by generation [69]. Our study has revealed that greater family size, and possibly its associated positive psychological well-being, may play a protective role against cancer initiation. The mechanisms may include following aspects:

1. Physiological and pathological functions of oxytocin in human health

Positive psychological well-being may make the functions of neuroendocrine and immune systems more efficient, which may reduce the risk of developing cancer [61, 70–72].

Oxytocin is a peptide hormone and neuropeptide. Its production is associated with good feelings and emotions [73]. Males and females can produce and release similar quantities of oxytocin [74] within the hypothalamo-pituitary magnocellular systems. Researches constantly revealed that family related activities are the major promotors of oxytocin production. A stream of studies in the last decade reported that oxytocin release is not only associated with giving birth [75] and lactation [76], but also with daily interactions between family members, such as spouses [33–35], mother and children [36], and father and children [37]. Oxytocin may be able to keep family happy and stable as it makes females and males stay monogamous [38, 39] and as it may bring positive psychological well-being to the family members. A self-reinforcing cycle is formed between family members interactions and oxytocin production.

Concurrently with the research into oxytocin production, physiological and pathological functions of oxytocin in humans have been the foci of numerous studies. Oxytocin has been postulated to have a role in inhibiting proliferation of human cancer cells, which may offer protective role in preventing cancer initiation [41]. The inhibitory role of oxytocin has been tested in individual site specific cancers, such as human breast cancer [41, 77] and ovarian cancer (animal model) [12]. A recent study reported that oxytocin, selectively activated by peptidylglycine α -amidating monooxygenase (PAM), may play a role in preventing and controlling a small cell lung cancer [78].

Bai et al. [27] reported that women with overall life satisfaction had less chance developing breast cancer. This may partly be true because life satisfaction may promote women to produce more oxytocin to prevent breast cancer cell initiation and proliferation. Another mechanism may be that greater TFR may make women produce less oestrogen and less menstrual cycles [10].

2. Less cancer genes/mutations accumulated in population with greater TFR/family size

Natural selection acts on each population [53, 79]. The total opportunity for natural selection in each population has been previously measured with the Biological State Index (I_bs_) [49, 50, 53–56, 80]. An I_bs_ value of one indicates total adaptation of the population to their environment. An I_bs_ value of zero signifies a total lack of adaptation (inability to overcome natural selection pressures that are present), and an impossibility to give life to the next generation [49, 50, 53–56, 80].

Our study indicated that Biological State Index (I_bs_) was in negative, strong and significant correlation to TFR/family size globally, in developed world and developing country groups respectively (Table 1). This means that population with greater TFR/family size is subject to more effective natural selection. As the consequence of less fitness, mortality rate due to various diseases, such as cancers, may increase [49, 50, 53–56, 79, 80]. Thus, cancer genes/mutations would be more often eliminated from a population with greater TFR/family size. Moreover, greater total fertility rates indicate less birth control therefore allowing more biological variation in fertility [81]. A portion of this variation, however small, provides opportunity for natural selection [81].

3. Family support and healthy lifestyle

Family members from the greater family size may interact with each other more often to create life satisfaction [43, 44]. Meanwhile, one family member can remind and/or recommend other members to have necessary medical examination and have a healthy lifestyle [42].

Bai et al. [27] reported that people with positive psychological well-being may practice healthy lifestyle, have the knowledge of cancer risks and benefits of regular physical examination. It was reported that such positive psychological well-being may decrease the risk in the development of breast cancer [27, 32, 82].

In this study, we have also observed in Fisher’s analysis that family size was in significantly stronger correlation with all cancers incidence in males (all ages) than it was with all cancers incidence in females (all ages). This finding is supported by the studies which found that males psychologically benefited more from having an extended kinship network than females [42, 63, 64]. However, this finding is inconsistent with Feller’s finding that reduced life satisfaction was more related to the development of cancer in women than in men [83]. The reason for this inconsistency might be that Feller’s data collection was based on the individual survey, which could be easily biased [31].

Family size has been implicated in the aetiologies of several individual site cancers, in previous studies based on the data collected at the individual level. Our findings were in agreement with the conclusions from the previous studies that greater family size was negatively correlated to the risks of developing bladder cancer [5], breast cancer [5, 6], colorectum cancer [5] and melanoma of skin. Although correlation does not necessarily imply causality, it may be suggested that increased family size may protect against the incidence of corpus uteri cancer and ovary cancer, but increase the risk of developing cervical cancer. These findings were in agreement with the prevailing dogma about the relationship between parity and gynecologic cancers, that is that more childbearing (greater TFR) may protect against corpus uteri cancer [8] and ovary cancer [9] due to less oestrogen production (less menstrual cycles) [10], but may contribute to cervix uteri cancer because of more exposure to infection risk [13]. However, our results were not supported by the findings from the study conducted by Hemminki et al. [5] that there were no reportable significant correlations between family size and risks of cervix uteri cancer, corpus uteri cancer and ovarian cancer. A number of studies have reported that ageing is one of the major contributors of corpus uteri cancer [84] and ovary cancer [42]. That findings of Hemminki et al. [5] were not compatible with our findings may be because only young females (aged mostly 5–43 years, up to 55 years) were included in their studies.

Poor hygiene level related infection with human papillomavirus is associated with cervical cancer initiation [85]. In the developed world, like Sweden, high level of hygiene or sanitation is accessible to almost all the residents. This reduces risk for females to have human papilloma virus infection, which may decrease the cervical cancer risk. This may be the explanation why Hemminki et al. [5] did not find the correlation between family size and cervical cancer incidence.

Blaser et al. [14] have reported that greater family size increased the risk of developing stomach cancer only for male family members, but not for all family members or female family members [14]. Aldrich et al. [15] reported that greater household size correlated with higher risk of lung cancer only in African Americans, but not in Latinos. However, sex specific or ancestry specific site cancer incidence was not included in our study. Thus, we may not be able to align our findings with the conclusions drawn by Blaser et al. [14] or Aldrich et al. [15].

The correlations, especially the partial correlations between family size and cancer variables in developing world were not as strong or significant as those identified in all countries (*n* = 178) and developed world (*n* = 98). This may be due to small variances (low standard deviations) of cancer incidence variables, which may reduce the covariance (correlation between family size and cancer variable), compared to those in the developed world and all countries grouping.

We must note an important strength of our study. Cancer risk studies based on surveys of individual persons have demonstrated a bias that is, in general, cancer patients tend to exaggerate negative life events in comparison to people with average or positive attitudes [31]. The methods employed in this study may have excluded this major bias because: 1) we used the objective measurement (TFR), instead of individual subjective psychological feeling assuming that TFR may be the family happiness index; 2) ecological study at population/group level, rather than individual based research method was adopted in this study. Ecological studies are based on aggregated quantitative data, not on the interviews with individual patients, so they are often used to determine the presence of effect of cancer risk-modifying factors in advance of, or impossible to identify in other epidemiological or laboratory approaches. Therefore, ecological study may be a better method to conduct the study of cancer incidence and its potential predictors, as cancer is one of the relatively rare diseases.

## Conclusions

Overall, this ecological study indicated that family size was negatively correlated with cancer incidence at population level. We also observed that family size correlated negatively with cancer incidence in males significantly stronger than with cancer incidence in females. Our results seem to suggest that it may be worthwhile further examining correlations between family size and cancer risk in males and females through the cohort and case-control studies based on large samples.

## Additional files

Additional file 1: The whole set of data for this study. (XLSX 47 kb)

Additional file 2: SD Fig. 1. The relationship between household size and all cancers incidence rates (total, male and female, all ages). (TIF 737 kb)

Additional file 3: **Table S1.** Pearson, Nonparametric and partial correlation between household size and each cancer variable and confounder. (DOCX 24 kb)

Additional file 4: **Table S2.** Stepwise multiple linear regression to identify the significant predictors of cancer incidence risk. (DOCX 21 kb)

Additional file 5: **Table S3.** Data descriptive and summary. (XLSX 16 kb)

## Abbreviations

FS: Family size
GDP: Gross Domestic Product
IARC: The International Agency for Research on Cancer of World Health Organization
TFR: Total fertility rate
UN: United Nations
WHO: World Health Organization
