# Supplementary figures and images for: Greater family size is associated with less cancer risk: an ecological analysis of 178 countries
Source: BMC Cancer. 2018 Sep 26;18:924. doi: 10.1186/s12885-018-4837-0 (PMC6156945; doi:10.1186/s12885-018-4837-0)

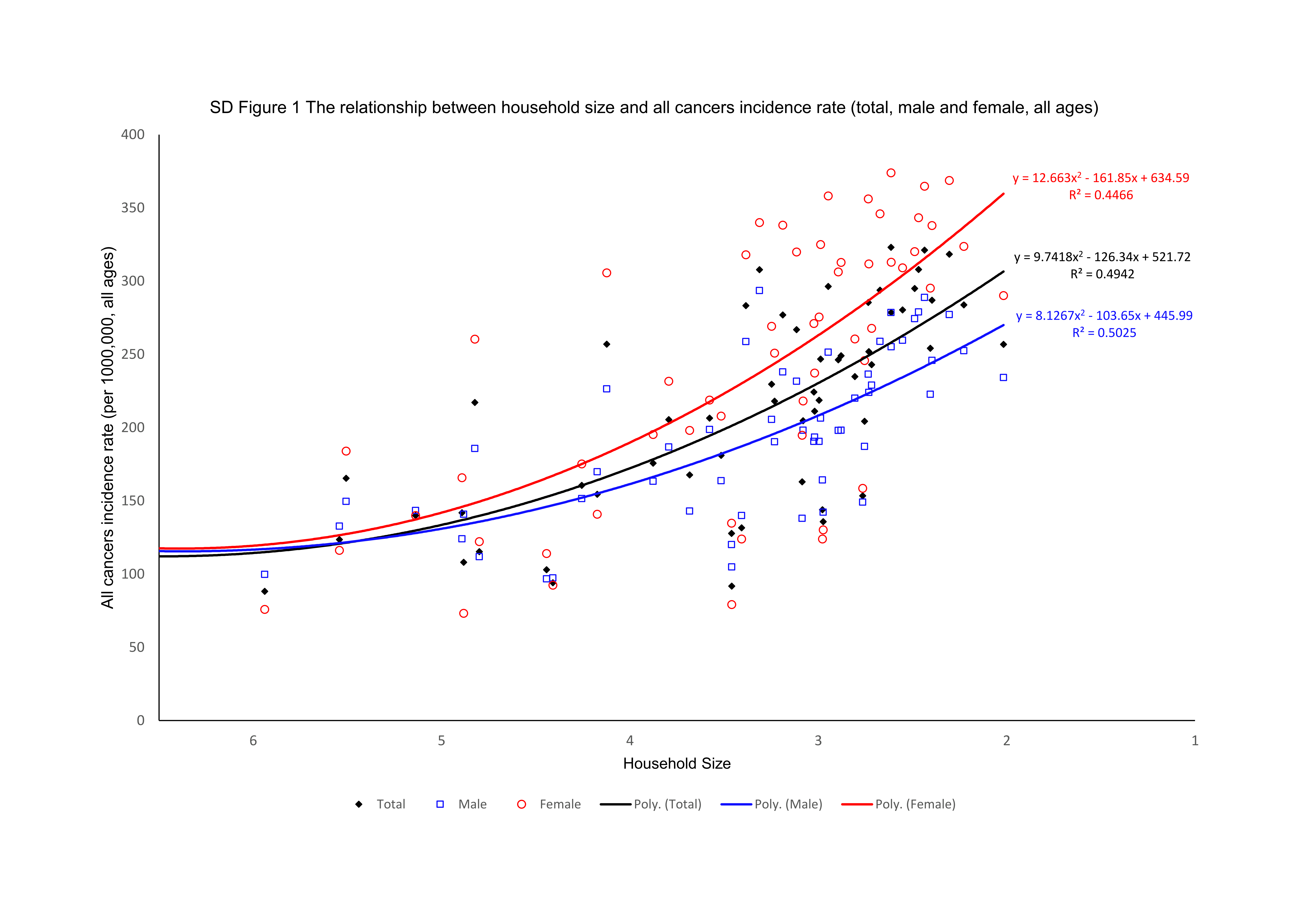

Supplement: Supplementary file 2 — SD Fig. 1. The relationship between household size and all cancers incidence rates (total, male and female, all ages). (TIF 737 kb) [file 12885_2018_4837_MOESM2_ESM.tif]
