# Supplementary material for: Greater family size is associated with less cancer risk: an ecological analysis of 178 countries
Source: BMC Cancer. 2018 Sep 26;18:924. doi: 10.1186/s12885-018-4837-0 (PMC6156945; doi:10.1186/s12885-018-4837-0)
Supplement: Supplementary file 3 — Table S1. Pearson, Nonparametric and partial correlation between household size and each cancer variable and confounder. (DOCX 24 kb) [file 12885_2018_4837_MOESM3_ESM.docx]

Table S1: Pearson, Nonparametric and partial correlation between household size and each cancer variable and confounder

|  | All countries, n=58 | | | | | | | |
| --- | --- | --- | --- | --- | --- | --- | --- | --- |
|  | Pearson | |  | Nonparametric | |  | Partial | |
|  | r | n |  | rho | n |  | r | df |
| All cancers excl. non-melanoma skin cancer (C00-97, but C44) - all ages: total | -0.635^***^ | 58 |  | -0.720^***^ | 58 |  | -0.492^***^ | 50 |
| All cancers excl. non-melanoma skin cancer (C00-97, but C44)- all ages: female | -0.624^***^ | 58 |  | -0.720^***^ | 58 |  | -0.482^***^ | 50 |
| All cancers excl. non-melanoma skin cancer (C00-97, but C44) - all ages: male | -0.593^***^ | 58 |  | -0.671^***^ | 58 |  | -0.429^*^ | 50 |
| All cancers excl. non-melanoma skin cancer (C00-97, but C44) – 0-49: total^ǂ^ | -0.629^***^ | 58 |  | -0.711^***^ | 58 |  | -0.493^***^ | 50 |
| All cancers excl. non-melanoma skin cancer (C00-97, but C44) – 0-49: female^ǂ^ | -0.586^***^ | 58 |  | -0.700^***^ | 58 |  | -0.422^**^ | 50 |
| All cancers excl. non-melanoma skin cancer (C00-97, but C44) – 0-49: male^ǂ^ | -0.617^***^ | 58 |  | -0.673^***^ | 58 |  | -0.486^***^ | 50 |
| Bladder (C67), all ages | -0.482^***^ | 58 |  | -0.593^***^ | 58 |  | -0.303^*^ | 50 |
| Breast(C50), all ages | -0.489^***^ | 58 |  | -0.672^***^ | 58 |  | -0.305^*^ | 50 |
| Cervix uteri (C53), all ages | 0.260^*^ | 58 |  | 0.331^*^ | 58 |  | 0.010 | 50 |
| Colorectum (C18-21), all ages | -0.508^***^ | 58 |  | -0.583^***^ | 58 |  | -0.278^*^ | 50 |
| Corpus uteri (C54), all ages | -0.493^***^ | 58 |  | -0.499^***^ | 58 |  | -0.324^*^ | 50 |
| Lung (C33-34), all ages | -0.380^**^ | 58 |  | -0.425^***^ | 58 |  | -0.059 | 50 |
| Melanoma of skin (C43), all ages | -0.646^***^ | 58 |  | -0.778^***^ | 58 |  | -0.564^***^ | 50 |
| Ovary (C56), all ages | -0.374^**^ | 58 |  | -0.456^***^ | 58 |  | -0.239 | 50 |
| Stomach (C16), all ages | -0.104 | 58 |  | 0.002 | 58 |  | 0.133 | 50 |
| GDP PPP 2010 | -0.463^***^ | 58 |  | -0.505^***^ | 58 |  | ^ | ^ |
| Urbanization 2010 | -0.388^**^ | 57 |  | -0.578^***^ | 57 |  | ^ | ^ |
| Life expectancy (e_60_, 2005-2010) | -0.346^**^ | 58 |  | -0.269^*^ | 58 |  | ^ | ^ |
| Biological State Index (I_bs_) | -0.400^**^ | 56 |  | -0.581^***^ | 56 |  | ^ | ^ |
| Note:  Pearson, Nonparametric and partial correlation reported. Significance level: *** p<0.001, ** p<0.01, * p<0.05  ^ Partial correlations were calculated when GDP, Urbanization, Life expectancy (e60) and Biological State Index (I_bs_) were kept statistically constant.  ^ǂ^ Life expectancy (e_50_) was not controlled for as it is not relevant in population segment aged 0-49 years old.  Data sources and variable meanings:  The International Agency for Research published cancer incidence rates (per 100,000 in 2012) of all cancers incidence rate by sex (total, male and female, 0-49 years and all ages respectively); bladder, breast, cervix uteri, colorectum, corpus uteri, ovary and stomach.  The World Bank data: GDP PPP (per capita purchasing power parity in current international $ in 2010) and Urbanization (the percentage of total population living urban areas in 2010)  The United Nations data: Life expectancy (e_60_, 2005-2010), the total population in households and the number of households for calculating household size. Household size is expressed as total number of persons in a household.  United Nations published (2008) country specific fertility data and WHO published (2012) life table were used for calculating the Biological State Index (I_bs_).  All variables were log-transformed for analysis in SPSS. | | | | | | | | |
