# Supplementary material for: Greater family size is associated with less cancer risk: an ecological analysis of 178 countries
Source: BMC Cancer. 2018 Sep 26;18:924. doi: 10.1186/s12885-018-4837-0 (PMC6156945; doi:10.1186/s12885-018-4837-0)
Supplement: Supplementary file 4 — Table S2. Stepwise multiple linear regression to identify the significant predictors of cancer incidence risk. (DOCX 21 kb) [file 12885_2018_4837_MOESM4_ESM.docx]

Table S2 Stepwise multiple linear regression to identify the significant predictors of cancer incidence risk

|  | All countries, n=58 | | | |
| --- | --- | --- | --- | --- |
|  | Rank | Predictor | Beta | Adjusted R^2^ |
| All cancers excl. non-melanoma skin cancer (C00-97, but C44) - all ages: both sexes | 1 | Life Expectancy | 0.302^**^ | 0.471 |
|  | 2 | Household Size | -0.362^***^ | 0.591 |
|  | 3 | Biological State Index (I_bs_) | 0.331^**^ | 0.645 |
| All cancers excl. non-melanoma skin cancer (C00-97, but C44)- all ages: female | 1 | Life Expectancy | 0.351^**^ | 0.479 |
|  | 2 | Household Size | -0.353^***^ | 0.589 |
|  | 3 | Biological State Index (I_bs_) | 0.273^*^ | 0.624 |
| All cancers excl. non-melanoma skin cancer (C00-97, but C44) - all ages: male | 1 | Biological State Index (I_bs_) | 0.317^*^ | 0.470 |
|  | 2 | Household Size | -0.343^***^ | 0.583 |
|  | 3 | GDP PPP | 0.319^*^ | 0.622 |
| All cancers excl. non-melanoma skin cancer (C00-97, but C44) – 0-49: both sexes^ǂ^ | 1 | GDP PPP | 0.512^***^ | 0.450 |
|  | 2 | Household Size | -0.431^***^ | 0.604 |
| All cancers excl. non-melanoma skin cancer (C00-97, but C44) – 0-49: female^ǂ^ | 1 | GDP PPP | 0.576^***^ | 0.503 |
|  | 2 | Household Size | -0.363^***^ | 0.601 |
| All cancers excl. non-melanoma skin cancer (C00-97, but C44) - all ages: male^ǂ^ | 1 | GDP PPP | 0.504^***^ | 0.435 |
|  | 2 | Household Size | -0.423^***^ | 0.582 |
| Note:  Significance level: *** p<0.001, ** p<0.01, * p<0.05  Variables (log-transformed) entered for multiple linear regression (stepwise) analysis: Household Size, Life Expectancy (e_60_), GDP PPP, Urbanization and Biological State Index (I_bs_).  ^ǂ^ Life expectancy (e_50_) was not included as it is not relevant in population segment aged 0-49 years old.  Data sources and variable meanings:  The International Agency for Research published cancer incidence rates (per 100,000 in 2012) of all cancers incidence rate by sex (total, male and female, 0-49 years and all ages respectively); bladder, breast, cervix uteri, colorectum, corpus uteri, ovary and stomach.  The World Bank data: Total Fertility Rate (the mean number of children born to a woman between 2009-2011), GDP PPP (per capita purchasing power parity in current international $ in 2010) and Urbanization (the percentage of total population living urban areas in 2010)  The United Nations data: Life expectancy (e_60_, 2005-2010), the total population in households and the number of households for calculating household size. Household size is expressed as total number of persons in a household.  United Nations published (2008) country specific fertility data and WHO published (2012) life table were used for calculating the Biological State Index (I_bs_). | | | | |
